# “Primary Omental Hydatid Cyst”: A Rare Entity

**DOI:** 10.1155/2012/654282

**Published:** 2012-09-23

**Authors:** Shailesh Sable, Jyoti Mehta, Sudeep Yadav, Priyadarshan Jategaokar, Premashish J. Haldar

**Affiliations:** ^1^Department of G.I Surgery, Jagjivanram Hospital, Maratha Mandir Lane, Mumbai 40008, India; ^2^Department of General Surgery, B.Y.L Nair Hospital, Mumbai 40008, India; ^3^Department of General Surgery, Jagjivanram Hospital, Maratha Mandir Lane, Mumbai 40008, India

## Abstract

Hydatid cyst is caused by the parasite *Echinococcus granulosus* commonly seen in temperate regions. Primary omental hydatid cyst is rare entity. Diagnosis can be achieved with contrast-enhanced computed tomography of abdomen and pelvis along with serology. Eosinophilia is a strong pointer to hydatid cyst as a differential diagnosis. Open or laparoscopic excision of the cyst along with medical therapy remains the treatment of choice.

## 1. Introduction

Hydatid disease is a parasitic disease that is caused by *Echinococcus granulosus*. It is a severe public health problem in the developing parts of the world. HD is commonly seen in liver and lungs but primary omental hydatid is rare.

## 2. Case Summary

**Figure 1:**
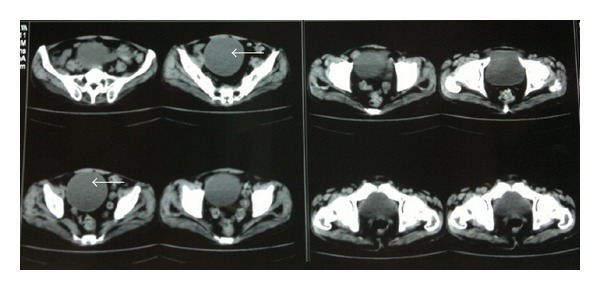
CECT of the pelvis shows solitary well-defined cystic lesion with clear fluid (WHO type CL) in pelvis.

A 52-year-old male presented with complaints of upper abdominal pain since one year on and off, which was dull aching nonradiating in the right hypochondrium. He also had occasional dyspepsia. However he had no history of jaundice, fever, or weight loss. Clinical examination did not reveal additional findings. Ultrasonography of abdomen and pelvis revealed cholelithiasis and a 10 × 9 cm solitary cystic lesion in the pelvis which had a well-defined wall with clear liquid. In view of this finding contrast-enhanced computed tomography of abdomen and pelvis was done which revealed a well-defined cyst in the pelvis arising from omentum and measuring approximately 10 × 9.5 cm with clear fluid inside and cholelithiasis (Figure 1). Liver, lungs, and other abdominal organs appeared to be normal. ELISA for *E. granulosus* was raised above the normal limits (patient value 6.8, normal value <1.1). Patient's complete blood counts were normal except eosinophilia. Liver function and renal function tests were within normal limits. 

**Figure 2:**
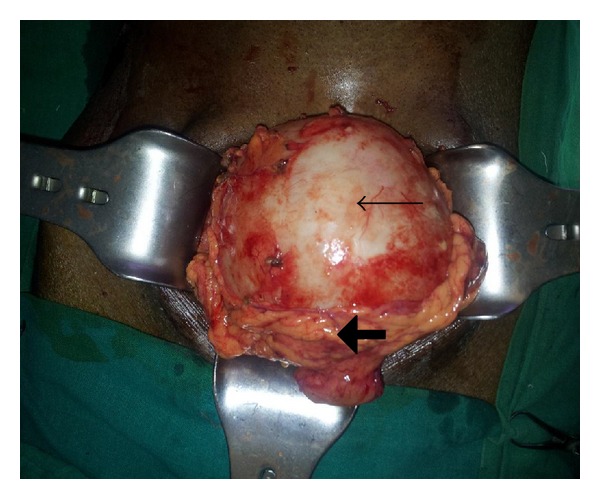
Omental hydatid cyst (thick arrow showing omentum, thin arrow showing hydatid cyst).

After preoperative course of Albendazole therapy, patient was planned for exploratory laparotomy. At laparotomy a 10 × 9 cm omental cyst was found without any infiltration into the surrounding structures. The cyst was excised in toto along with the rim of omentum all around (Figure 2). Open cholecystectomy was also added in view of symptomatic gall stones. Exploration revealed no other pathology/cyst in the abdomen. Postoperative course of the patient was uneventful. Histopathology report of the cyst revealed germinal layers with scolices along with necrosis and inflammation of cyst wall. He was not advised postoperative medical therapy in view of complete excision of the cyst without any spillage of the cyst contents. On followup at one year he is symptom-free and recurrence-free.

## 3. Discussion

Hydatid cyst is caused by the parasite *Echinococcus granulosus* commonly seen in temperate regions. The adult worm resides in Dog or Wolf's intestine (definitive host). Definite host shed eggs in their stool which contaminate vegetables and fruits. These eggs are then ingested by the cattle or sheep during grazing in the fields. Humans are intermediate and accidental host. Humans are infected by eating contaminated vegetables or fruits. These parasite oncospheres after entering the stomach or intestine start penetrating their wall and reach liver parenchyma through portal circulation. After reaching the liver which acts as a filter for the parasite (dead end), larval stage development begins (cyst formation). Some of the oncospheres may bypass liver and reach lungs through systemic circulation and form larval stage there. Hydatid cyst commonly affects liver and lungs. Primary omental hydatid cyst without liver and lung involvement is rare. Intraperitoneal hydatid disease is seen in about 3.9–12.5% of patients [1, 2]. One of the theories cited for possibility of primary omental hydatid is spillage from ruptured hydatid cyst of liver, and it is difficult to prove that it has developed secondary to the liver [3].

A role of lymphatic system in seeding of oncospheres directly from the bowel to the site of development of intra-abdominal cyst has also been shown [4]. Primary omental hydatid cyst may be asymptomatic and can get diagnosed incidentally as in our case. It may also present as lump in the abdomen or symptoms due to compression of the surrounding viscera. Differential diagnosis of the intra-abdominal cystic lesions arising from omentum includes mesenteric cyst, gastrointestinal duplication cyst, ovarian cysts, cystadenoma, and lymphangioma. If the cyst is complicated, then the differential diagnosis should also include intra-abdominal abscess, hematoma, and loculated ascites [1]. Specifically from a country like India where abdominal tuberculosis is very commonly seen, abdominal cystic lesion should be treated with caution. Ultrasonography of the abdomen is the initial imaging to identify the organ of origin and to characterise the cyst. However a contrast enhanced computed tomography is always required to confirm the diagnosis as well as to plan the therapy [5]. ELISA can be a good serological test for the confirmation of hydatid cyst with a sensitivity of 95–97%. Parasitic infestation may also present with asymptomatic rise in the eosinophil counts as seen in our patient. Prompt treatment of these cysts is recommended as they are prone to complications like rupture, haemorrhage, infection, or torsion [6]. It is also wise to rule out abdominal tuberculosis as it may change the plan of treatment. There are various case reports published on isolated or primary omental or peritoneal hydatid cyst from all over the world [1, 3, 6–8], but very few has been reported from Indian continent [2, 9].

The ideal treatment for omental hydatid is controlled open surgical excision as there is a very high possibility of uncontrolled spillage and peritoneal dissemination of the disease leading to recurrence [9, 10]. Laparoscopic cyst excision although technically difficult can be attempted with due risk of spillage if expertise for the same is available [11]. Special precautions should be taken like covering the rest of the peritoneal cavity with betadine or 20% saline soaked sponges to avoid implantation of the cyst in case of rupture.

 In conclusion, primary omental hydatid cyst can occur without affecting other viscera. Hydatid cyst should be kept as one of the differential diagnosis for any patient presenting with a cystic lump in the abdomen especially from endemic areas. Eosinophilia on peripheral smear may also help in supporting the diagnosis of parasitic infestation especially in the setting of the diagnostic dilemma. Abdominal tuberculosis should always be ruled out before planning a surgical therapy. Open or laparoscopic excision of the cyst should be performed in a suspected hydatid cyst in the omentum to prevent recurrence.

## References

[1] Sekmenli T, Koplay M, Sezgin A (2009). Isolated omental hydatid cyst: clinical, radiologic, and pathologic findings. *Journal of Pediatric Surgery*.

[2] Sethi SK, Patnaik S, Narayan, Nayak SN (2004). Isolated omental hydatid cyst—a case report. *Journal of the Indian Medical Association*.

[3] Wani RA, Malik AA, Chowdri NA, Wani KA, Naqash SH (2005). Primary extrahepatic abdominal hydatidosis. *International Journal of Surgery*.

[4] Morris DL, Richards KS (1992). *Biology of Echinococcus. Hydatid Disease. Current Medical and Surgical Management*.

[5] Ilica AT, Kocaoglu M, Zeybek N (2007). Extrahepatic abdominal hydatid disease caused by Echinococcus granulosus: imaging findings. *American Journal of Roentgenology*.

[6] Uramatsu M, Saida Y, Nagao J (2001). Omental cyst: report of a case. *Surgery Today*.

[7] Annandale T (1877). Case of large hydatid tumour of the Omentum treated successfully by a free incision, with antiseptic precautions. *British Medical Journal*.

[8] Alis H, Kapan S, Öner O (2009). Primary omental hydatid cyst. *International Medical Case Reports Journal*.

[9] Rathod KJ, Lyndogh S, Kanojia RP, Rao KL (2011). Multiple primary omental hydatid: rare site for a common infestation. *Tropical Gastroenterology*.

[10] Filippou D, Tselepis D, Filippou G, Papadopoulos V (2007). Advances in Liver Echinococcosis: diagnosis and Treatment. *Clinical Gastroenterology and Hepatology*.

[11] Bickel A, Loberant N, Singer-Jordan J, Goldfeld M, Daud G, Eitan A (2001). The laparoscopic approach to abdominal hydatid cysts: a prospective nonselective study using the isolated hypobaric technique. *Archives of Surgery*.

